# Supplementary material for: Modelling to explore the potential impact of asymptomatic human infections on transmission and dynamics of African sleeping sickness
Source: PLoS Comput Biol. 2021 Sep 13;17(9):e1009367. doi: 10.1371/journal.pcbi.1009367 (PMC8459990; doi:10.1371/journal.pcbi.1009367)
Supplement: S1 Methods — (PDF) [file pcbi.1009367.s001.pdf]

Supplementary materials: S1 Methods

Modelling to explore the potential impact of asymptomatic human infections on transmission and dynamics of African sleeping sickness

Maryam Aliee[1,2*], Matt J. Keeling[1,2,3], Kat S. Rock[1,2]
[1]Mathematics Institute, University of Warwick, Coventry, UK
[2]Zeeman Institute for Systems Biology and Infectious Disease Epidemiology Research, University of Warwick, Coventry, UK
[3]School of Life Sciences, University of Warwick, Coventry, UK

1 Estimating parameters of the baseline gHAT model

As described in previous versions of the original deterministic model [1,2], there are estimates available for some parameters of the model in the literature. These parameters are normally assigned by fixed values as they are unlikely to vary much geographically or in time, however, we consider a biologically plausible range for each in our sensitivity analysis.

The other parameter values were taken from posterior distributions by fitting the model to data. In this approach, the deterministic model was fitted to health-zone-level data for different health zones of the Democratic Republic of Congo (DRC) using an adaptive Metropolis-Hastings MCMC algorithm [2]. For the fitting process in the previous study, informative priors were used based on estimates or prior knowledge about parameters. Moreover, fitting the model to the province-level data provided priors for some parameters, particularly relating to treatment rates from stages 1 and 2, γ_H^{post} and η_H^{post}, and improvement in passive detection rates $\eta_{H\text{amp}}$, $\gamma_{H\text{amp}}$, and d_{steep}.

In our sensitivity analysis, we used these previous priors to guide our parameter ranges used in our sensitivity anlaysis and expanded some of them to account for possible fluctuation since the model structure has now been changed. In the rest of the manuscript, when a specific parameter set was needed, we chose the most likely parameter sets from the posterior distributions for the moderate-endemicity health zone of Mosango in Kwilu province, DRC.

2 Model comparison

We summarise a comparison of the structures of our minimal asymptomatic model and the baseline model in Table 1. As stated in the main text, we assume that exposed people develop either blood parasite infection, I_{1H}^b, with probability p_{bs}, or skin-only infection, I_{1H}^s, with probability $1 - p_{bs}$. We add the possibility of self-cure for both the skin-only and blood parasite groups through the parameters ω_H^b for blood and ω_H^s for skin-only.

Table 1: Comparison between the modelling assumptions of the asymptomatic model and the baseline model as in [2].

	**Asymptomatic model**	**Baseline model**
**Possibility of blood infection**	Included<br>$E_H \xrightarrow{p_{bs}\sigma_H} I^b_{1H} \quad p_{bs} < 1$<br>with self-cure possibility<br>$\omega^b_H > 0$<br>The rate of progression from E_H to any infection type is the same as baseline	Included<br>$E_H \xrightarrow{\sigma_H} I^b_{1H}$<br>no self-cure possibility<br>$\omega^b_H = 0$
**Possibility of skin infection**	Included<br>$E_H \xrightarrow{(1-p_{bs})\sigma_H} I^s_{1H} \quad p_{bs} < 1$<br>Skin-only infectiousness realtive to blood infection<br>$0 < x \leq 1$	Not included<br>$p_{bs} = 1$
**Active screening possibility to detect infection**	Stage 2: ✓<br>Stage 1 blood infection:✓<br>Stage 1 skin-only infection: ×	Stage 2:✓<br>Stage 1:✓
**Passive screening possibility to detect infection**	Stage 2: ✓<br>Stage 1 blood infection:✓after 1998 but at a lower rate than stage 2<br>Stage 1 skin-only infection: ×	Stage 2:✓<br>Stage 1:✓after 1998 but at a lower rate than stage 2
**Model fitting**	Not done in the current version	Fitted to 2000–2016 data across health zones of DRC
**Animal reservoir**	Not included	Not included

3 Endemic equilibrium of the asymptomatic model

We calculate the endemic equilibrium of our asymptomatic model by balancing all time derivatives in dynamic equations

$$
\begin{aligned}
\alpha m_{\text{eff}} f(i)\frac{S_{Hi}}{N_{Hi}} I_V &= (\sigma_H+\mu_H)E_{Hi},\\
I^s_{1Hi} &= \frac{(1-p_{bs})}{(\omega^s_H+\theta_I+\mu_H)}\sigma_H E_{Hi},\\
I^b_{1Hi} &= \frac{p_{bs}\sigma_H E_{Hi}+\theta_I I^s_{1Hi}}{(\varphi_H+\mu_H+\eta_H+\omega^b_H)} = \frac{p_{bs}(\omega^s_H+\mu_H)+\theta_I}{(\varphi_H+\mu_H+\eta_H+\omega^b_H)(\omega^s_H+\theta_I+\mu_H)}\sigma_H E_{Hi},\\
I_{2Hi} &= \frac{\varphi_H}{(\gamma_H+\mu_H)} I^b_{1Hi},\\
p_{Hi} &= \frac{\eta_H I^b_{1Hi}+\gamma_H I_{2Hi}}{(\omega_H+\mu_H)}\\
&= \frac{1}{(\omega_H+\mu_H)}(\eta_H+\frac{\gamma_H\varphi_H}{\gamma_H+\mu_H})\frac{p_{bs}(\omega^s_H+\mu_H)+\theta_I}{(\varphi_H+\mu_H+\eta_H+\omega^b_H)(\omega^s_H+\theta_I+\mu_H)}\sigma_H E_{Hi},\\
S_{Hi} &= N_{Hi}+[\omega_H p_{Hi}+\omega^b_H I^b_{1Hi}+\omega^s_H I^s_{1Hi}-\alpha m_{\text{eff}} f(i)\frac{S_{Hi}}{N_{Hi}} I_V]/\mu_H\\
&= N_{Hi}+\frac{\sigma_H}{\mu_H}[\frac{\omega_H}{\omega_H+\mu_H}(\eta_H+\frac{\gamma_H\varphi_H}{\gamma_H+\mu_H})\frac{p_{bs}(\omega^s_H+\mu_H)+\theta_I}{(\varphi_H+\mu_H+\eta_H+\omega^b_H)(\omega^s_H+\theta_I+\mu_H)}\\
&+ \frac{\omega^s_H(1-p_{bs})}{\omega^s_H+\theta_I+\mu_H}+\frac{\omega^b_H(p_{bs}(\omega^s_H+\mu_H)+\theta_I)}{(\varphi_H+\mu_H+\eta_H+\omega^b_H)(\omega^s_H+\theta_I+\mu_H)}-\frac{\mu_H}{\sigma_H}-1]E_{Hi}\\
&= N_{Hi}+AE_{Hi}. \qquad (3.1)
\end{aligned}
$$

And for vectors:

$$
\begin{aligned}
S_V &= \frac{\mu_V}{\mu_V+\alpha}N_V,\\
E_{2V} &= \frac{3\sigma_V}{(3\sigma_V+\mu_V)}E_{1V},\\
E_{3V} &= \frac{3\sigma_V}{(3\sigma_V+\mu_V)}E_{2V},\\
I_V &= \frac{3\sigma_V}{\mu_V}E_{3V} = \frac{(3\sigma_V)^3}{(3\sigma_V+\mu_V)^2\mu_V}E_{1V} = BE_{1V}, \qquad (3.2)
\end{aligned}
$$

and also

$$
\begin{aligned}
(3\sigma_V+\mu_V)E_{1V} &= \alpha p_V\sum_i f(i)\frac{(I^b_{1Hi}+x_1 I^s_{1Hi}+I_{2Hi})}{N_{Hi}}(S_V+\varepsilon G_V),\\
G_V &= \frac{\alpha}{\mu_V}S_V-(\frac{3\sigma_V}{\mu_V}+1)E_{1V}. \qquad (3.3)
\end{aligned}
$$

In this framework, the dynamics of pupal stage is interpolated within susceptible population dynamics, requiring $B_V = \frac{\mu_V}{\mathbb{P}(\text{pupating})}(1+\frac{\mu_V N_H}{K\xi_V^2\mathbb{P}(\text{pupating})})$.

Using $S_V+\varepsilon G_V = (1+\frac{\varepsilon\alpha}{\mu_V})S_V-(\frac{3\sigma_V}{\mu_V}+1)\varepsilon E_{1V}$ and the notation $I^b_{1Hi}+x_1 I^s_{1Hi}+I_{2Hi} = CE_{Hi}$

$$
EE_{1V} = C\alpha p_V\sum_i f(i)\frac{E_{Hi}}{N_{Hi}}\left((1+\frac{\varepsilon\alpha}{\mu_V})S_V-\varepsilon(\frac{3\sigma_V}{\mu_V}+1)E_{1V}\right). \qquad (3.4)
$$

Here $E = 3\sigma_V + \mu_V$. Now, we just need to replace

$$E_{Hi} = \frac{I_V f(i) \alpha m_{\text{eff}}}{D - I_V A \alpha m_{\text{eff}} f(i)/N_{Hi}} = \frac{E_{1V} a f(i)}{D - E_{1V} b f(i)/N_{Hi}}, \tag{3.5}$$

and therefore

$$E = B\,(G - F E_{1V}) \sum_i \frac{a f^2(i)/N_{Hi}}{D - b E_{1V} f(i)/N_{Hi}}, \tag{3.6}$$

where

$$B = \frac{(3\sigma_V)^3}{(3\sigma_V + \mu_V)^2 \mu_V}\ , \qquad G = C\alpha p_V N_V \frac{\mu_V + \varepsilon\alpha}{\mu_V + \varepsilon}\ , \qquad F = C\alpha p_V \varepsilon(\frac{3\sigma_V}{\mu_V} + 1),$$
$$a = B\alpha m_{\text{eff}}\ , \qquad b = AB\alpha m_{\text{eff}}\ , \qquad D = \sigma_H + \mu_H\ , \tag{3.7}$$

Considering two groups of low and high risk populations with corresponding indices of 1 and 4, we can solve the 2nd order polynomial equation to calculate E_{1V} (3rd order but with a zero solution)

$$\begin{aligned} E_{1V}^2 \frac{f(1)f(4)}{N(1)N(4)} \left(Eb^2 - Fab[f(1) + f(4)]\right) \\ +E_{1V} \left(-EDb[\frac{f(1)}{N(1)} + \frac{f(4)}{N(4)}] + Gab\frac{f(1)f(4)}{N(1)N(4)}[f(1) + f(4)] + FaD[\frac{f^2(1)}{N(1)} + \frac{f^2(4)}{N(4)}]\right) \\ +ED^2 - GDa\left(\frac{f^2(1)}{N(1)} + \frac{f^2(4)}{N(4)}\right) = 0 \end{aligned} \tag{3.8}$$

Estimating reproduction ratio

The new generation matrix is given by

$$K = -T\Sigma^{-1}. \tag{3.9}$$

Transmission tensor:

$$T = \left(\begin{array}{cccccccccccccc} & & & & & & & & & & & & & \alpha p_H f(1) \\ & & & & & & & & & & & & & 0 \\ & & & & & & & & & & & & & 0 \\ & & & & & & & & & & & & & 0 \\ & & & & & & & & & & & & & \alpha p_H f(4) \\ & & & & & & 0 & & & & & & & 0 \\ & & & & & & & & & & & & & 0 \\ & & & & & & & & & & & & & 0 \\ & & & & & & & & & & & & & \alpha p_H f(A) \\ & & & & & & & & & & & & & 0 \\ 0 & x_1 t\frac{f(1)}{N_{H1}} & t\frac{f(1)}{N_{H1}} & t\frac{f(1)}{N_{H1}} & 0 & x_1 t\frac{f(1)}{N_{H4}} & t\frac{f(4)}{N_{H4}} & t\frac{f(4)}{N_{H4}} & 0 & t\frac{f(A)}{N_A} & 0 & 0 & 0 & 0 \\ & & & & & & & & & & & & & 0 \\ & & & & & & 0 & & & & & & & 0 \\ & & & & & & & & & & & & & 0 \end{array}\right) \tag{3.10}$$

Here $t = \alpha p_V N_V \frac{\mu_V + \varepsilon\alpha}{\mu_V + \alpha}$.

Transition tensor

$$\Sigma = \begin{pmatrix} \Sigma_H & 0 & 0 & 0 \\ 0 & \Sigma_H & 0 & 0 \\ 0 & 0 & \Sigma_A & 0 \\ 0 & 0 & 0 & \Sigma_V \end{pmatrix}, \tag{3.11}$$

with

$$
\begin{aligned}
\Sigma_H &= \begin{pmatrix} -\sigma_H - \mu_H & 0 & 0 & 0 \\ (1-p_{bs})\sigma_H & -\omega_H^s - \theta_I - \mu_H & 0 & 0 \\ p_{bs}\sigma_H & \theta_I & -\varphi_H - \eta_H - \omega_H^b - \mu_H & 0 \\ 0 & 0 & \varphi_H & -\gamma_H - \mu_H \end{pmatrix}, \\
\Sigma_A &= \begin{pmatrix} -\sigma_A - \mu_A & 0 \\ \sigma_A & -\varphi_A - \mu_A \end{pmatrix}, \\
\Sigma_V &= \begin{pmatrix} -3\sigma_V - \mu_V & 0 & 0 & 0 \\ 3\sigma_V & -3\sigma_V - \mu_V & 0 & 0 \\ 0 & 3\sigma_V & -3\sigma_V - \mu_V & 0 \\ 0 & 0 & 3\sigma_V & -3\sigma_V - \mu_V \end{pmatrix}.
\end{aligned} \tag{3.12}
$$

R_0 is given as the biggest eigenvalue of K.

References

[1] Rock KS, Torr SJ, Lumbala C, Keeling MJ. Quantitative evaluation of the strategy to eliminate human African trypanosomiasis in the DRC. Parasites & Vectors. 2015;8(1):532.

[2] Crump RE, Huang CI, Knock E, Spencer SEF, Brown PE, Mwamba Miaka E, et al. Quantifying epidemiological drivers of gambiense human African Trypanosomiasis across the Democratic Republic of Congo. 2020;doi:10.1101/2020.06.23.20138065.
